# Maternal smoking during pregnancy and risk of childhood-onset type 1 diabetes in offspring: A systematic review and meta-analysis

**DOI:** 10.17305/bb.2025.13063

**Published:** 2025-11-10

**Authors:** Shu Zhang, Lishi Zhao, Jiao Li

**Affiliations:** 1 Department of Endocrinology, Chongqing Youyoubaobei Women and Children’s Hospital, Chongqing, China; 2 Department of Pediatrics, Chongqing Youyoubaobei Women and Children’s Hospital, Chongqing, China

**Keywords:** Type 1 diabetes, children, smoking, pregnancy, risk factor

## Abstract

Childhood-onset type 1 diabetes (T1D) is a chronic autoimmune disease characterized by a steadily increasing global incidence and significant public health implications. The relationship between maternal smoking during pregnancy and T1D risk remains uncertain. To clarify this association, we conducted a meta-analysis of prospective cohort studies to enhance methodological reliability. We systematically searched PubMed, Embase, and Web of Science from their inception to May 2025 for prospective cohort studies examining the link between maternal smoking during pregnancy and the incidence of T1D in offspring. Risk ratios (RRs) and 95% confidence intervals (CIs) were pooled using a random-effects model, accounting for heterogeneity. Twelve prospective cohort datasets from ten studies, encompassing over 5.9 million children, were included. Maternal smoking during pregnancy was significantly associated with a reduced risk of childhood-onset T1D (RR: 0.74, 95% CI: 0.72–0.76, *P* < 0.001), with no evidence of statistical heterogeneity (*I*^2^ ═ 0%, *P* ═ 0.48). This association remained robust across sensitivity analyses that excluded one dataset at a time. Subgroup analyses demonstrated consistent results across various categories, including cohort size, prevalence of maternal smoking, method of T1D diagnosis, and adjustments for maternal age, diabetes, and delivery mode. Notably, the inverse association was significantly weaker in studies that did not adjust for maternal diabetes (RR: 0.79 vs 0.72, *P* for subgroup difference = 0.01). We found no substantial evidence of publication bias (Egger’s test, *P* ═ 0.55). In conclusion, this meta-analysis identified an inverse association between maternal smoking during pregnancy and the incidence of childhood-onset T1D. However, this finding should be interpreted cautiously, as residual confounding cannot be ruled out, and maternal smoking is associated with numerous serious adverse health consequences.

## Introduction

Type 1 diabetes (T1D) is a chronic autoimmune disorder characterized by the destruction of pancreatic β-cells, resulting in lifelong insulin dependence [1, 2]. The global incidence of childhood-onset T1D has been steadily rising, particularly in developed nations, presenting a significant public health challenge due to its considerable burden on patients, families, and healthcare systems [3]. Children diagnosed with T1D face risks of acute metabolic complications, long-term microvascular and macrovascular complications, and psychosocial stress, underscoring the necessity for early prevention strategies [4–6]. Although genetic predisposition is a significant factor, emerging evidence indicates that environmental exposures during early life also play a vital role in the development of T1D [7]. Established maternal and perinatal risk factors include advanced maternal age, maternal diabetes, and cesarean delivery; however, these factors account for only a fraction of the variability in disease presentation [8–10].

Maternal smoking during pregnancy is a prevalent exposure, with estimates ranging from 9% to over 50% in certain populations [11]. Smoking during gestation is known to affect fetal growth and development [12] and has been linked to various adverse outcomes in offspring, including low birth weight [13], respiratory illnesses [14], and neurodevelopmental disorders [15]. Maternal smoking may influence the risk of childhood-onset T1D through several potential mechanisms. Nicotine and other constituents of tobacco smoke possess immunomodulatory properties that could alter fetal immune system development and increase susceptibility to autoimmune diseases [11, 16, 17]. These biological considerations, combined with inconsistent epidemiological findings [18], underscore the necessity for a systematic evaluation of this association. A previous meta-analysis suggested an inverse relationship between maternal smoking during pregnancy and offspring T1D risk; however, this conclusion was primarily based on retrospective studies and exhibited significant heterogeneity, raising concerns regarding its validity [18]. Given the accumulation of large, population-based prospective cohort studies in recent years [19–23], a re-evaluation utilizing methodologically robust data is warranted. Thus, the present study aimed to comprehensively assess the relationship between maternal smoking during pregnancy and the incidence of childhood-onset T1D in offspring by synthesizing evidence exclusively from prospective cohort studies. By limiting inclusion to studies with prospective exposure assessment and longitudinal outcome follow-up, we sought to minimize bias and provide more reliable evidence to inform public health understanding and future research on prenatal risk factors for T1D.

## Materials and methods

The study was conducted in accordance with the PRISMA 2020 guidelines [24, 25] and the Cochrane Handbook [26] for Systematic Reviews of Interventions, ensuring methodological rigor in study selection, data extraction, statistical analysis, and result interpretation. The protocol was prospectively registered on PROSPERO with the registration ID CRD420251116685.

### Literature search

A comprehensive literature search was conducted in PubMed, Embase, and Web of Science, employing a broad set of search terms that integrated the following keywords and concepts: (1) “pregnant” OR “pregnancy” OR “prenatal” OR “pre-natal”; (2) “smoking” OR “smoke” OR “cigarette” OR “cigarettes” OR “nicotine” OR “tobacco”; (3) “diabetes” OR “diabetic” OR “type 1 diabetes” OR “T1D” OR “T1DM”; and (4) “child” OR “children” OR “adolescent” OR “adolescents” OR “pediatric” OR “paediatric” OR “offspring” OR “childhood” OR “adolescence.” The search was restricted to human studies and included only full-text articles published in English in peer-reviewed journals. To ensure completeness, we also manually screened the reference lists of relevant original and review articles for additional eligible studies. The search encompassed all publications from database inception up to May 25, 2025.

### Study eligible criteria

We applied the PICOS framework to define the inclusion criteria: Population (P): Children (aged 0–18 years) born to mothers with documented smoking status during pregnancy.

Intervention/Exposure (I): Maternal smoking during pregnancy (any trimester), as assessed by self-report, medical records, or biomarker validation.

Comparator (C): Children born to non-smoking mothers during pregnancy.

Outcome (O): Incidence of childhood-onset T1D, diagnosed by clinical or registry-based criteria.

Study design (S): Prospective cohort studies with follow-up from birth to ascertain incident T1D in offspring, published as full-length articles in peer-reviewed journals.

Studies were excluded if they were reviews, editorials, meta-analyses, retrospective cohort or case-control studies, cross-sectional studies, studies reporting maternal smoking outside of pregnancy or passive smoking, or studies including adult-onset T1D in offspring. In cases of overlapping populations, only the study with the largest sample size was retained for inclusion in the meta-analysis.

### Study quality evaluation

Two reviewers independently conducted the literature search, screened studies, assessed methodological quality, and extracted data. Discrepancies were resolved through consultation with the corresponding author. The quality of the included studies was evaluated using the Newcastle–Ottawa scale (NOS) [27], which examines study selection, control of confounding variables, and outcome assessment. The NOS assigns scores ranging from 1 to 9, with a score of 8 or above indicating high methodological quality.

### Data collection

The data collected for the meta-analysis included study details (author, year, and study country), participant characteristics (source of the cohort, number of children in each study, and sex distribution), exposure details (methods for evaluating maternal smoking during pregnancy, number of children born to mothers who smoked during the index pregnancy), age of children (range and mean) for the diagnosis of T1D, methods for diagnosing T1D, number of children who developed T1D, and covariates adjusted for in the regression models.

### Statistical analysis

Risk ratios (RRs) and 95% confidence intervals (CIs) were used to assess the association between maternal smoking during pregnancy and the risk of childhood-onset T1D in offspring. RRs and their standard errors were either directly extracted or derived from reported 95% CIs or *P* values, followed by logarithmic transformation to stabilize variance and achieve a normal distribution [26]. If multiple RRs were reported from different models, we selected the one with the most comprehensive adjustment. Heterogeneity was assessed using the Cochrane *Q* test and the *I*^2^ statistic [28], with a *P* value < 0.10 indicating significant heterogeneity, and *I*^2^ values of <25%, 25%–75%, and >75% indicating low, moderate, and high heterogeneity, respectively. A random-effects model was employed to synthesize the data, accommodating variability across studies [26]. We utilized a random-effects model for all meta-analyses to account for potential clinical heterogeneity across studies (e.g., differences in populations, exposure definitions, and study periods), even when statistical heterogeneity was minimal (τ^2^ ═ 0, *I*^2^ ═ 0%). To assess the stability of the results, sensitivity analyses were conducted by sequentially excluding each study. Additionally, subgroup analyses were performed to evaluate the impact of predefined study characteristics on the meta-analysis results, including sample size, prevalence of maternal smoking during pregnancy in each study, method for diagnosing T1D (clinical diagnosis vs database codes), incidence of T1D in children from each study, and adjustment for potential confounding factors such as maternal age, maternal diabetes, and delivery type. For continuous study-level variables, subgroup analyses were stratified at the median to provide balanced comparisons in the absence of universally accepted clinical cut-offs. Publication bias was evaluated through funnel plot visualization and assessed for asymmetry using Egger’s regression test [29]. All analyses were performed using RevMan (Version 5.3; Cochrane Collaboration, Oxford, UK) and Stata (Version 17.0; Stata Corporation, College Station, TX, USA).

## Results

### Study inclusion

**Figure 1.**
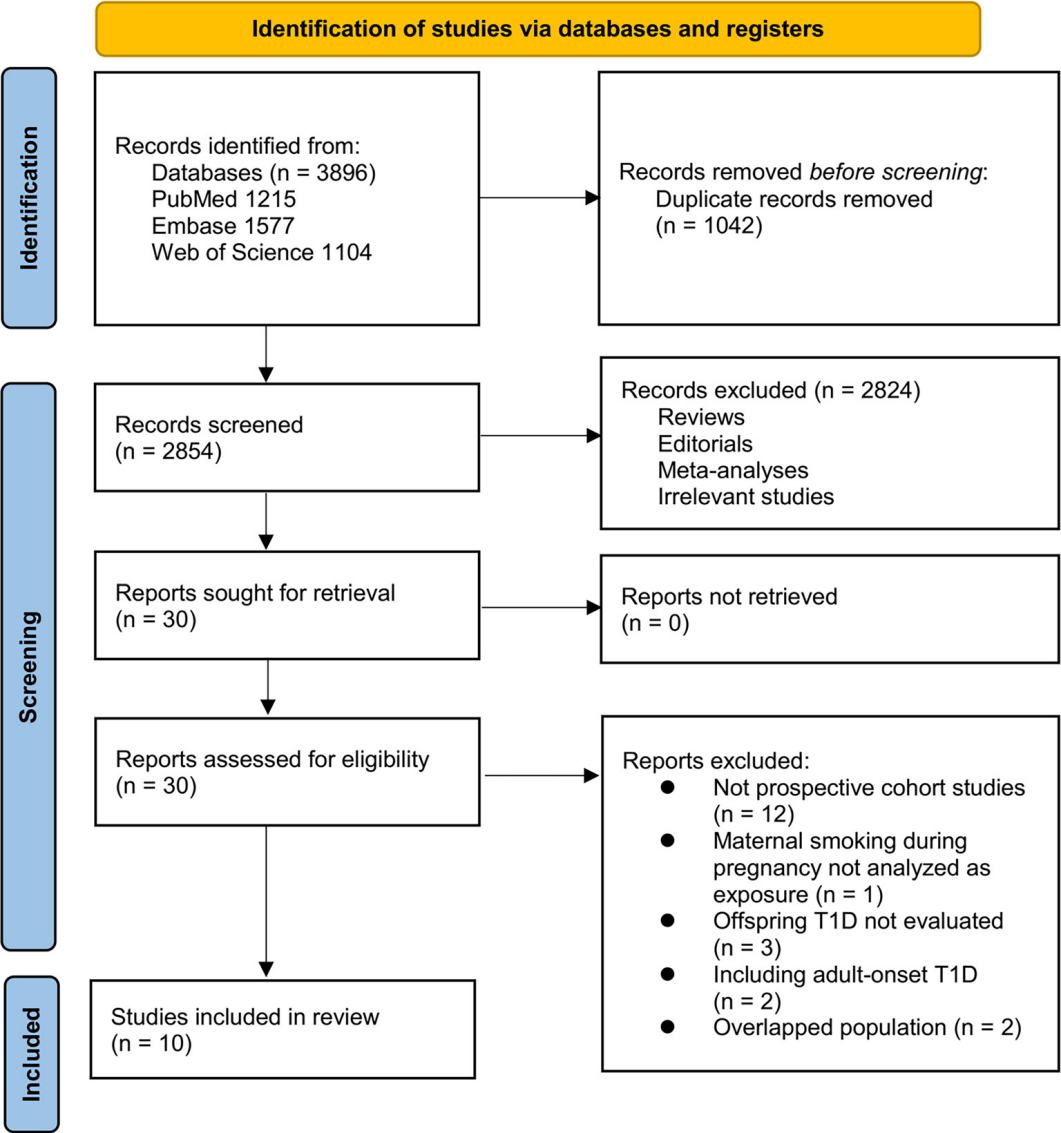
**Flowchart of database search and study inclusion**.

The study selection process is depicted in Figure 1. We initially identified 3896 records from three databases. After removing 1042 duplicate records, 2854 articles underwent title and abstract screening. Of these, 2824 were excluded for not aligning with the objectives of the meta-analysis. The remaining 30 full-text articles were assessed independently by two reviewers, resulting in the exclusion of 20 studies for specific reasons detailed in Figure 1. Ultimately, 10 studies were included in the subsequent analysis [19–23, 30–34].

**Table 1:** Characteristics of the included prospective studies

**Study**	**Country**	**Cohort source**	**No. of children included**	**Male (%)**	**Methods for validation of MSDP**	**No. of children born to MSDP**	**Age at T1D diagnosis (years)**	**Methods for the diagnosis of T1D**	**No. of children with T1D**	**Variables adjusted or controlled**	**Prevalence of MSDP (%)**	**Incidence of T1D in offspring (%)**
Frederiksen, 2013	USA	Prospective birth cohort of children at increased genetic risk for T1D	1835	48.7	Maternal self-report during pregnancy	183	<18 (mean: 5.9)	Physician-diagnosed T1D based on symptoms (polyuria/polydipsia) + random glucose ≥ 200 mg/dL or OGTT (fasting ≥ 126 mg/dL or 2h ≥ 200 mg/dL)	53	Age, sex, HLA genotype, first-degree relative with T1D, maternal education, and delivery type	10.0	2.89
Haynes, 2014	Australia	Population-based birth cohort to mother without diabetes in West Australia	226,233	NR	Maternal self-report during pregnancy	40,876	<15 (mean: NR)	Physician-diagnosed based on clinical, biochemical, and autoantibody criteria	287	Age, sex, maternal socioeconomic status, birthweight, gestational age, maternal age, birth order, and year of birth	18.1	0.13
Adlercreutz, 2015	Sweden	Population-based birth cohort (singleton births)	768,395	51.4	Maternal self-report at first antenatal visit	176,044	<14 (median: 4.7)	Hospital registry (ICD codes)	4518	Age, sex, maternal age, small for gestational age, delivery mode, preterm birth, season of birth, maternal birth country, congenital malformations, socioeconomic factors	22.9	0.59
Lund-Blix, 2015	Norway	Prospective birth cohort of children at increased genetic risk for T1D	726	50.6	Maternal smoking during pregnancy reported via questionnaire at child’s age 3 months.	116	<12 (mean: 7.7)	National Childhood Diabetes Registry (clinical diagnosis)	25	Age, sex, first-degree relative with T1D, vitamin D supplementation, maternal education, and delivery type	16.0	3.44
Hussen, 2015	Sweden	Population-based birth cohort (Migration and Health Cohort)	1,176,155	NR	Maternal self-report at first antenatal visit	136,719	<18 (mean: 7.8)	National Patient Register (ICD codes)	5771	Age, sex, birth cohort, maternal diabetes, paternal diabetes, maternal BMI, maternal age, gestational age, parental education, mode of delivery	11.6	0.49
Magnus, 2018 MoBa	Norway	Prospective pregnancy cohort (1999–2008)	98,287	51	Maternal self-reported at gestational week 18; cord blood cotinine measured in a subset (*n* ═ 630)	26098	<15 (median: 7.1)	National Childhood Diabetes Registry (clinical diagnosis)	349	Age, sex, maternal age, parity, education, prepregnancy BMI, diabetes; HLA genotype in subset	26.6	0.36
Magnus, 2018 DNBC	Denmark	Prospective pregnancy cohort (1996–2002)	86,785	51	Maternal self-reported at gestational week 12	26,262	<17 (median: 10.1)	National Childhood Diabetes Registry (clinical diagnosis)	340	Age, sex, maternal age, parity, education, prepregnancy BMI, diabetes	30.3	0.39
Magnus, 2018 NRBC	Norway	National registry linkage	434,627	NR	Maternal self-reported at delivery	245,999	<11 (mean: 4.7)	Patient registry (ICD codes)	692	Age, sex, maternal age, parity, education, insulin-treated diabetes	56.6	0.16
Begum, 2020	Australia	Population-based birth cohort in South Australia	286,058	NR	Maternal self-reported at first antenatal visit (<20 weeks) and second half of pregnancy (≥20 weeks)	62,216	<15 (mean: NR)	Patient registry (ICD codes)	557	Age, sex, maternal birth region, ethnicity, remoteness, socioeconomic status, hospital category, parity, and pre-pregnancy hypertension/diabetes	21.7	0.19
Metsälä, 2020	Finland	Population-based birth cohort	134,078	51.3	Maternal self-reported during first trimester	20,378	<16 (mean: 7.1)	Patient registry (ICD codes)	6862	Age, sex, maternal age, diabetes, asthma; birth decade, gestational age, birth weight/length, mode of delivery, parity, and socioeconomic factors	15.2	5.12
Raisanen, 2021	Finland	Nationwide register-based cohort study	11,407	52.2	Maternal self-reported during first trimester	1048	<17 (mean: 8.6)	Patient registry (ICD codes)	102	Age, sex, maternal age, employment, parity; gestational age, birthweight, delivery method, postnatal antibiotics	9.2	0.89
Wei, 2023	Sweden	Nationwide Swedish registers	3,170,386	NR	Maternal self-reported smoking status at first prenatal visit (8–12 weeks of pregnancy).	NR	<18 (mean: NR)	Patient registry (ICD codes)	18,745	Age, sex, calendar year, family history of diabetes, maternal BMI, and parental education	NR	0.59

The study conducted by Magnus (2018) reported on three cohorts that were independently included in the meta-analysis: the Norwegian Mother and Child Cohort Study (MoBa), the Danish National Birth Cohort (DNBC), and the Norwegian Registry Birth Cohort (NRBC). The data presented by Metsälä et al. (2020) reflects the case-cohort design of the Finnish registry study, which included all 6,862 children diagnosed with type 1 diabetes alongside a 10% random sample of 127,216 children without the condition. Consequently, the proportion of cases reported should not be interpreted as the population incidence but rather as a characteristic of the study design. Abbreviations: MSDP: Maternal smoking during pregnancy; T1D: Type 1 diabetes; OGTT: Oral glucose tolerance test; ICD: International Classification of Diseases; NR: Not reported.

**Table 2:** Study quality evaluation via the Newcastle–Ottawa scale

**Study**	**Representativeness of the exposed cohort**	**Selection of the non-exposed cohort**	**Ascertainment of exposure**	**Outcome not present at baseline**	**Control for age**	**Control for other confounding factors**	**Assessment of outcome**	**Enough long follow-up duration**	**Adequacy of follow-up of cohorts**	**Total**
Frederiksen, 2013	1	1	1	1	1	1	1	1	1	9
Haynes, 2014	1	1	1	1	1	1	1	1	1	9
Adlercreutz, 2015	1	1	1	1	1	1	0	1	1	8
Lund-Blix, 2015	1	1	1	1	1	1	1	1	1	9
Hussen, 2015	1	1	1	1	1	1	0	1	1	8
Magnus, 2018 MoBa	1	1	1	1	1	1	1	1	1	9
Magnus, 2018 DNBC	1	1	1	1	1	1	1	1	1	9
Magnus, 2018 NRBC	1	1	1	1	1	1	0	1	1	8
Begum, 2020	1	1	1	1	1	1	0	1	1	8
Metsälä, 2020	1	1	1	1	1	1	0	1	1	8
Raisanen, 2021	1	1	1	1	1	1	0	1	1	8
Wei, 2023	1	1	1	1	1	1	0	1	1	8

The 2018 study conducted by Magnus reported on three cohorts that were independently incorporated into the meta-analysis: the Norwegian Mother and Child Cohort Study (MoBa), the Danish National Birth Cohort (DNBC), and the Norwegian Registry Birth Cohort (NRBC).

### Summary of study characteristics

Overall, ten prospective cohort studies were included in the meta-analysis [19–23, 30–34]. Notably, the study by Magnus (2018) [19] reported three independent cohorts, which were separately included in the meta-analysis: the Norwegian Mother and Child Cohort Study (MoBa), the Danish National Birth Cohort (DNBC), and the Norwegian Registry Birth Cohort (NRBC), resulting in a total of 12 datasets. The characteristics of the 12 prospective cohort datasets included in this meta-analysis are summarized in Table 1. These studies were conducted in the United States, Australia, Sweden, Norway, Denmark, and Finland, and were published between 2013 and 2023. All studies employed a prospective design, enrolling large, population-based or high-risk birth cohorts to examine the association between maternal smoking during pregnancy and childhood-onset T1D. Sample sizes varied widely, ranging from 726 to 3,170,386 children. Maternal smoking during pregnancy was primarily assessed via self-report at various time points, including the first prenatal visit, gestational weeks 12–18, or through postnatal questionnaires; one study also used cord blood cotinine for partial validation [19]. The diagnosis of T1D was based on clinical criteria [19, 30, 31, 34] or national diabetes or patient registries using International Classification of Diseases (ICD) codes [19–23, 32, 33]. All studies reported the incidence of childhood-onset T1D, with diagnoses occurring before 18 years of age. The number of children diagnosed with T1D ranged from 25 to 18,745 per study. In the study by Metsälä et al. (2020) [21], all 6862 T1D cases and a 10% random reference cohort of 127,216 non-cases were analyzed, yielding a total of 134,078 children. The relatively high proportion of cases (5.1%) arises from this case–cohort sampling scheme rather than the underlying population incidence. All studies adjusted for key confounding variables, typically including child’s age and sex, maternal age, socioeconomic status, parity, delivery mode, and family history of diabetes, to varying extents. The prevalence of mothers who smoked during the index pregnancy ranged from 9.2% to 56.6%, while the incidence of T1D among children in each study ranged from 0.13% to 5.12%. Study quality was evaluated using the NOS (Table 2), with total scores ranging from 8 to 9, indicating consistently high methodological quality across all studies. Five datasets received the maximum score of 9 [19, 30, 31, 34], reflecting excellent cohort representativeness, exposure and outcome ascertainment, and adequate follow-up. The remaining seven datasets scored 8 [19–23, 32, 33], primarily due to reliance on ICD codes rather than clinical evaluation for diagnosis. Nevertheless, the overall robustness of exposure definition, longitudinal outcome assessment, and adjustment for major confounders enhances the validity of the synthesized findings in this meta-analysis.

**Figure 2.**
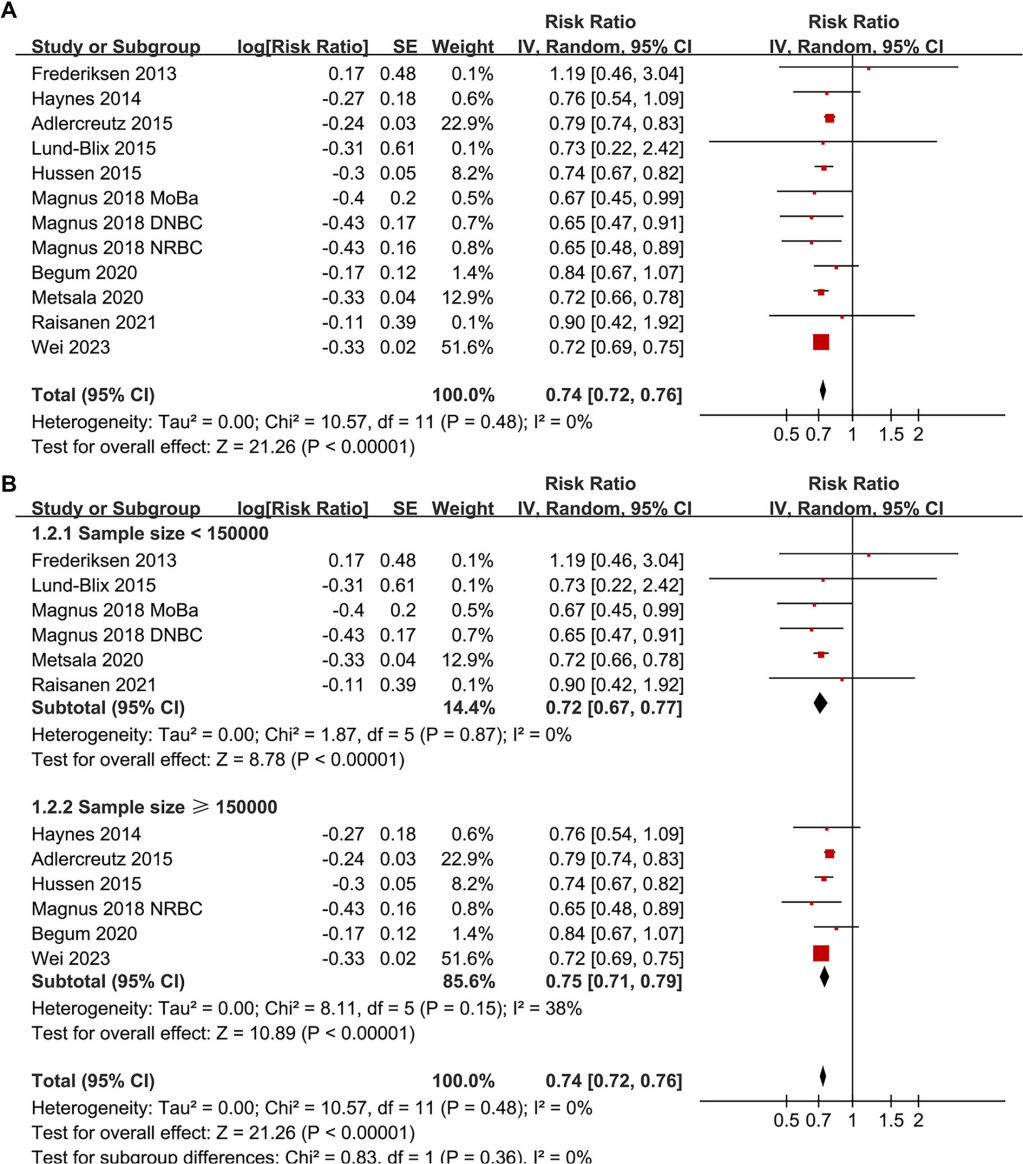
**Forest plots for the meta-analysis of the association between maternal smoking during pregnancy and the risk of childhood-onset T1D in offspring.** (A) Overall meta-analysis; (B) Subgroup analysis based on the samples of included studies. RRs with 95% CIs are presented for each study. Weights are derived from inverse-variance random-effects models. Abbreviations: T1D: Type 1 diabetes; RR: Risk ratio; CI: Confidence interval; SE: Standard error; df: Degrees of freedom; τ^2^: Between-study variance; *I*^2^: Percentage of total variation due to heterogeneity; Chi^2^: Cochran’s Q statistic.

**Table 3:** Sensitivity analysis by excluding one dataset at a time

**Dataset excluded**	**RR (95% CI)**	***I* ^2^**	***P* for Cochrane *Q* test**	***P* for effect**
Frederiksen, 2013	0.74 [0.72, 0.76]	0%	0.48	<0.001
Haynes, 2014	0.74 [0.72, 0.76]	5%	0.40	<0.001
Adlercreutz, 2015	0.72 [0.70, 0.75]	0%	0.93	<0.001
Lund-Blix, 2015	0.74 [0.72, 0.76]	5%	0.39	<0.001
Hussen, 2015	0.74 [0.71, 0.76]	5%	0.39	<0.001
Magnus, 2018 MoBa	0.74 [0.72, 0.76]	3%	0.41	<0.001
Magnus, 2018 DNBC	0.74 [0.72, 0.76]	0%	0.44	<0.001
Magnus, 2018 NRBC	0.74 [0.72, 0.76]	0%	0.44	<0.001
Begum, 2020	0.74 [0.71, 0.76]	0%	0.51	<0.001
Metsälä, 2020	0.74 [0.72, 0.76]	1%	0.43	<0.001
Raisanen, 2021	0.74 [0.72, 0.76]	3%	0.41	<0.001
Wei, 2023	0.76 [0.73, 0.79]	0%	0.648	<0.001

The 2018 study conducted by Magnus included three independent cohorts in its meta-analysis: the Norwegian Mother and Child Cohort Study (MoBa), the Danish National Birth Cohort (DNBC), and the Norwegian Registry Birth Cohort (NRBC).

### Association between maternal smoking in pregnancy and childhood-onset T1D

**Figure 3.**
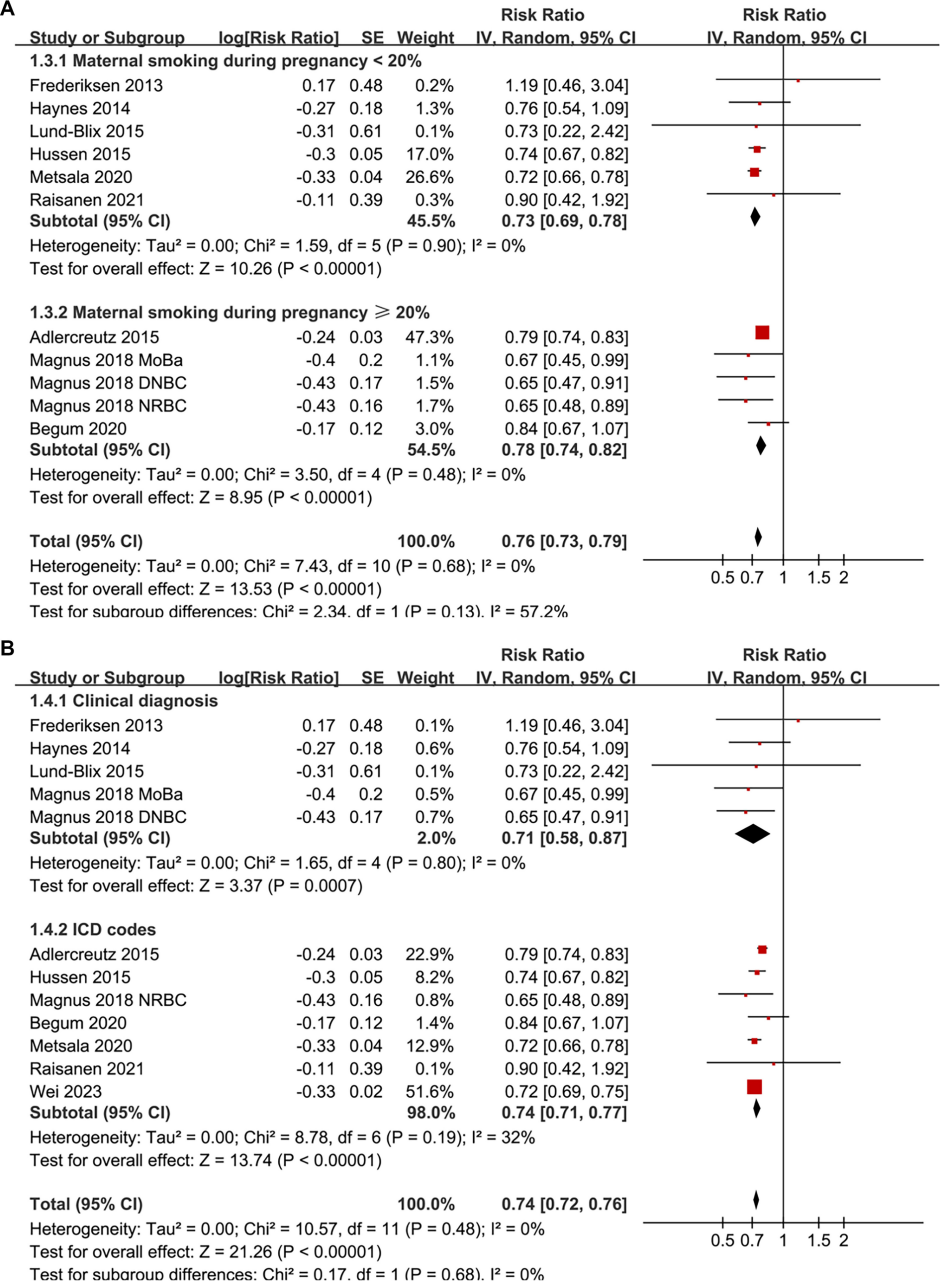
**Forest plots for the subgroup analyses of the association between maternal smoking during pregnancy and the risk of childhood-onset T1D in offspring.** (A) Subgroup analysis based on the prevalence of maternal smoking during pregnancy across each study; (B) Subgroup analysis according to diagnostic methods for T1D. RRs with 95% CIs are presented for each study. Weights are calculated using inverse-variance random-effects models. Abbreviations: T1D: Type 1 diabetes; RR: Risk ratio; CI: Confidence interval; SE: Standard error; df: Degrees of freedom; τ^2^: Between-study variance; *I*^2^: Percentage of total variation due to heterogeneity; Chi^2^: Cochran’s Q statistic.

**Figure 4.**
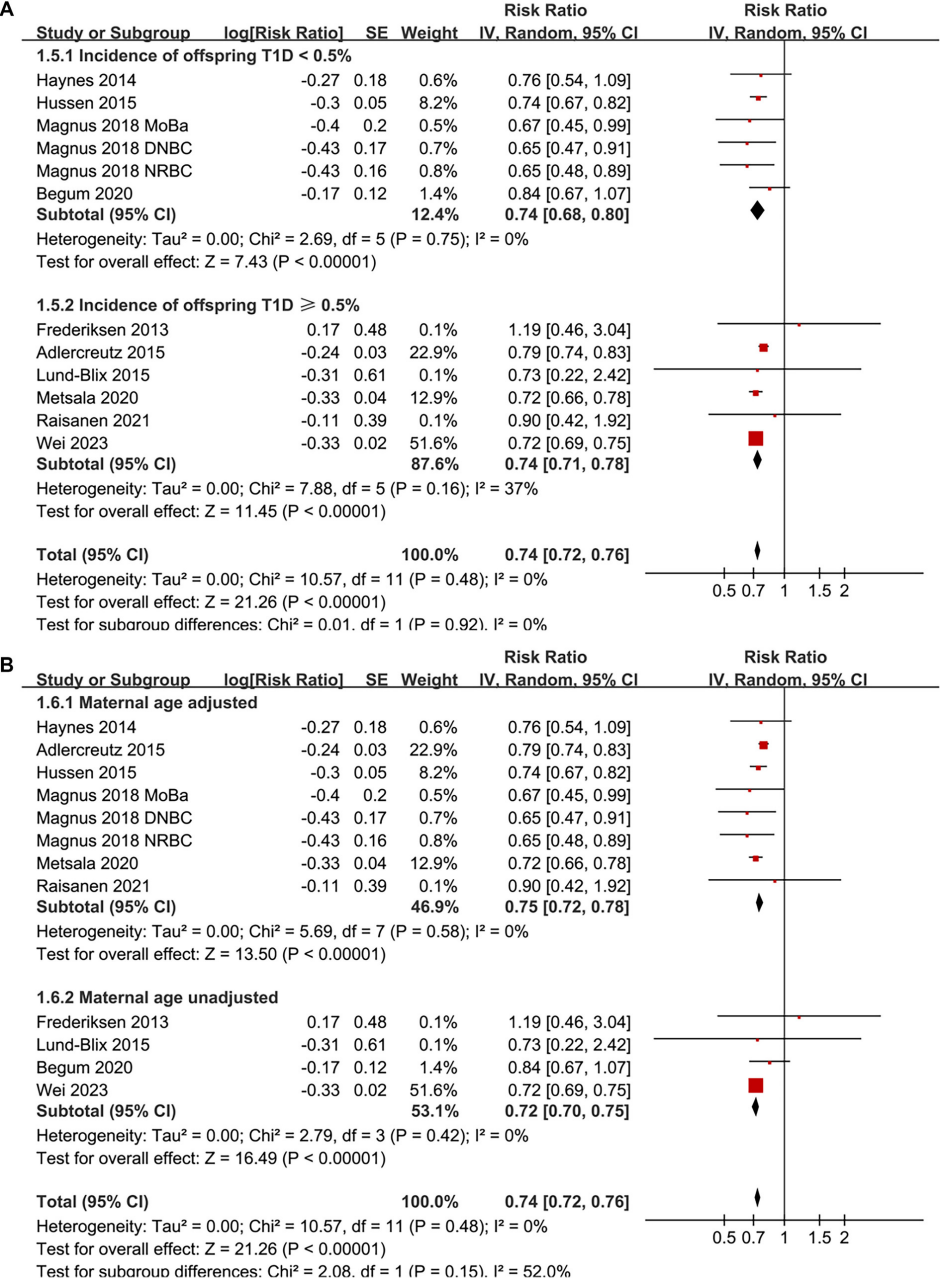
**Forest plots for the subgroup analyses of the association between maternal smoking during pregnancy and the risk of childhood-onset T1D in offspring.** (A) Subgroup analysis based on the incidence of offspring T1D in each study; (B) Subgroup analysis based on whether maternal age was adjusted in each study. RRs with 95% CIs are presented for each study. Weights are calculated using inverse-variance random-effects models. Abbreviations: T1D: Type 1 diabetes; RR: Risk ratio; CI: Confidence interval; SE: Standard error; df: Degrees of freedom; τ^2^: Between-study variance; *I*^2^: Percentage of total variation due to heterogeneity; Chi^2^: Cochran’s Q statistic.

**Figure 5.**
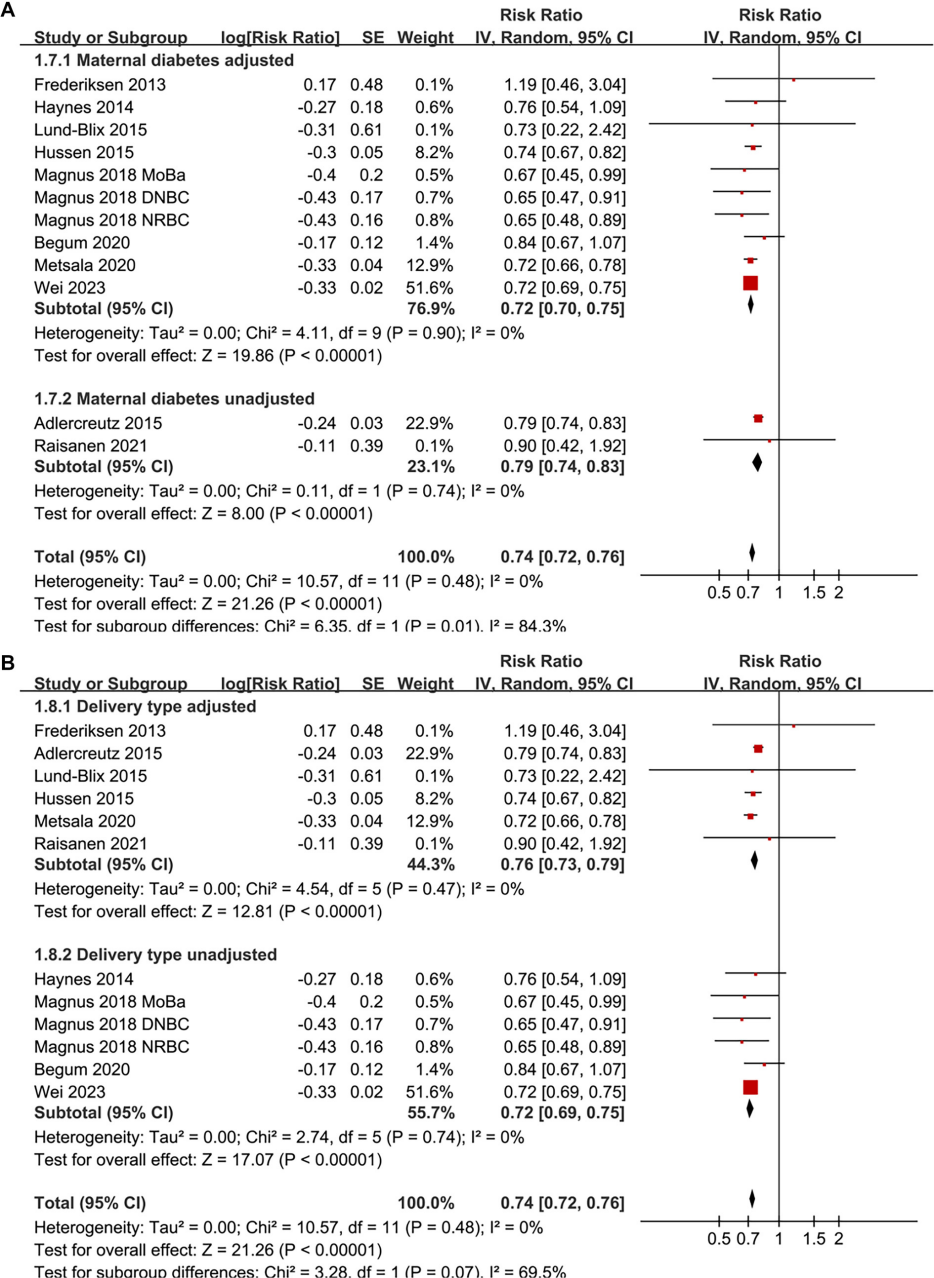
**Forest plots for the subgroup analyses of the association between maternal smoking during pregnancy and the risk of childhood-onset T1D in offspring.** (A) Subgroup analysis based on the adjustment for maternal diabetes in each study; (B) Subgroup analysis based on the adjustment for delivery type in each study. RRs with 95% CIs are presented for each study. Weights are calculated using inverse-variance random-effects models. Abbreviations: T1D: Type 1 diabetes; RR: Risk ratio; CI: Confidence interval; SE: Standard error; df: Degrees of freedom; τ^2^: Between-study variance; *I*^2^: Percentage of total variation due to heterogeneity; Chi^2^: Cochran’s Q statistic.

Pooled results from a random-effects model, which included 12 prospective cohort datasets, indicated that maternal smoking during pregnancy is significantly associated with a reduced incidence of childhood-onset T1D in offspring (RR: 0.74, 95% CI: 0.72–0.76, *P* < 0.001; Figure 2A). No significant heterogeneity was detected (*P* for the Cochrane *Q* test = 0.48; *I*^2^ ═ 0%). Sensitivity analyses, conducted by removing one dataset at a time, demonstrated stable results (RR: 0.72–0.76, *P* < 0.05 for all comparisons; Table 3). Further subgroup analyses revealed that the association was consistent across studies with sample sizes < or ≥ 150,000 (RR: 0.72 vs 0.75; *P* for subgroup difference = 0.36; Figure 2B), across studies where the prevalence of maternal smoking during pregnancy was < or ≥ 20% (RR: 0.73 vs 0.78; *P* for subgroup difference = 0.13; Figure 3A), in studies diagnosing T1D via clinical evaluation or ICD codes (RR: 0.71 vs 0.74; *P* for subgroup difference = 0.68; Figure 3B), and between studies where the incidence of T1D in offspring was < or ≥ 0.5% (RR: 0.74 vs 0.74; *P* for subgroup difference = 0.92; Figure 4A). Additionally, subgroup results were significant for analyses with and without adjustments for maternal age (RR: 0.75 vs 0.72; *P* for subgroup difference = 0.15; Figure 4B), maternal diabetes status (RR: 0.72 vs 0.79; *P* for subgroup difference = 0.01; Figure 5A), and delivery type (RR: 0.76 vs 0.72; *P* for subgroup difference = 0.07; Figure 5B).

### Publication bias

**Figure 6.**
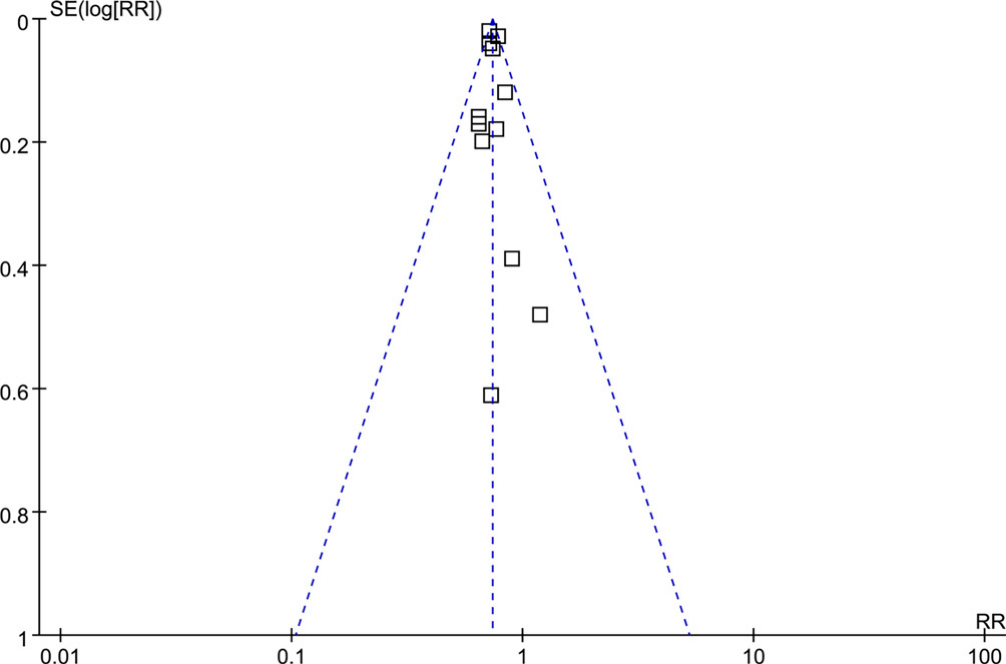
**Funnel plots for assessing potential publication biases in meta-analyses of maternal smoking during pregnancy and the risk of childhood-onset T1D in offspring.** Each dot represents an individual dataset, while the vertical line denotes the pooled effect estimate. The symmetry of the plot suggests an absence of small-study effects; however, formal tests may lack power due to the inclusion of only 12 datasets. All 12 studies are represented, and some markers overlap due to nearly identical coordinates of effect estimates and standard errors. Abbreviation: T1D: Type 1 diabetes.

Funnel plots assessing the relationship between maternal smoking during pregnancy and the risk of childhood-onset T1D in offspring are presented in Figure 6. The visual symmetry of these plots suggests a low likelihood of publication bias. Furthermore, Egger’s test did not provide strong evidence of publication bias (*P* ═ 0.55).

## Discussion

This meta-analysis of 12 prospective cohort datasets offers robust evidence regarding the relationship between maternal smoking during pregnancy and the risk of childhood-onset T1D in offspring. By including over 5.9 million children from well-characterized cohorts across multiple countries, the findings reveal a consistent inverse association between maternal smoking during pregnancy and the subsequent development of T1D in children. The absence of heterogeneity across studies and the stability of results in sensitivity analyses bolster the reliability of this observed association.

Several potential biological mechanisms may elucidate how maternal smoking during pregnancy influences the risk of T1D in offspring. Nicotine, a major component of cigarette smoke, readily crosses the placenta and may modulate fetal immune development by altering cytokine expression and immune tolerance, potentially dampening autoimmune responses later in life [35, 36]. Additionally, prenatal exposure to smoking has been linked to increased levels of regulatory T cells and reduced pro-inflammatory responses in offspring, which may offer protection against autoimmune diseases such as T1D [37, 38]. Epigenetic modifications, including DNA methylation changes induced by tobacco exposure, may also contribute to the reprogramming of immune pathways and pancreatic β-cell development, although the precise mechanisms warrant further investigation [16, 39].

The subgroup analyses provide crucial insights into the robustness and potential modifiers of the observed association. The inverse relationship persisted across studies regardless of sample size, prevalence of maternal smoking, and diagnostic methods for T1D, suggesting that these factors do not significantly influence the findings. Notably, the association appeared weaker in studies that did not adjust for maternal diabetes, underscoring the importance of controlling for confounding maternal metabolic factors. Similarly, adjustments for maternal age and delivery mode did not substantially alter the results, indicating that these variables may not be major confounders in this context. These findings reinforce the consistency of the inverse association across various study settings and populations.

This meta-analysis has several strengths. First, it is the most comprehensive and up-to-date synthesis of prospective cohort studies addressing this research question, surpassing earlier meta-analyses that included retrospective designs and mixed-age outcomes. By restricting inclusion to prospective studies with prenatal exposure assessment and longitudinal follow-up, the risk of recall bias, selection bias, and reverse causality is minimized [40]. Second, all included studies utilized multivariable regression models, adjusting for a wide range of relevant confounders, such as child age and sex, maternal age, diabetes status, socioeconomic factors, parity, and delivery mode, thereby enhancing the validity of the findings. Third, the consistently high quality of the included studies, as assessed by the NOS, adds to the credibility of the results. Lastly, the large cumulative sample size ensures sufficient power to detect associations and facilitates informative subgroup and sensitivity analyses.

Nevertheless, several limitations must be considered when interpreting these findings. Although all studies adjusted for multiple confounders, the possibility of residual confounding from unmeasured or inaccurately measured factors cannot be excluded. For instance, maternal lifestyle behaviors, genetic predispositions, and exposure to various environmental factors may influence both smoking behavior and the risk of T1D in offspring [41, 42]. Moreover, maternal smoking exposure was primarily assessed via self-report, which may introduce misclassification bias; however, this bias is likely non-differential and would generally attenuate the observed associations. Only one study incorporated biomarker validation through cord blood cotinine [19], highlighting the need for future research to utilize more objective measures of exposure. Additionally, while the studies uniformly focused on childhood-onset T1D, variations in diagnostic criteria or registry accuracy may still exist, although subgroup analyses by diagnostic method did not reveal significant heterogeneity. A further limitation is that most studies did not provide information on smoking intensity (e.g., cigarettes per day) or trimester-specific exposure, which precluded a dose–response meta-analysis. Future research should aim to obtain more detailed exposure data to elucidate potential dose–response relationships. Furthermore, although the contour-enhanced funnel plot did not suggest substantial publication bias, the limited number of available datasets constrains the reliability of such assessments. Therefore, the possibility of publication bias cannot be ruled out, and the results should be interpreted with caution. Finally, the observational nature of the included studies limits any inference of causality, and the counterintuitive direction of the association necessitates careful interpretation.

From a clinical and public health perspective, these findings do not suggest that maternal smoking should be encouraged during pregnancy. The well-documented adverse effects of prenatal tobacco exposure on fetal growth, neurodevelopment, and respiratory health outweigh any potential protective association with T1D. Rather, these results may indicate underlying biological pathways activated by tobacco exposure, which could inspire mechanistic studies focused on immune modulation and β-cell preservation. Understanding these pathways may ultimately inform preventive or therapeutic strategies that mimic the immunoregulatory effects without the harmful consequences of smoking. In this context, the observed inverse association may serve as a foundation for future research rather than a clinical recommendation. Future studies are needed to explore the biological plausibility and mechanistic basis of this association. Prospective cohorts with biomarker-confirmed exposure data and detailed immune phenotyping of offspring would be particularly valuable. Investigating gene–environment interactions, including maternal and fetal genotypes related to immune regulation, xenobiotic metabolism, and nicotine sensitivity, may clarify whether specific subpopulations are more susceptible to the protective or harmful effects of prenatal smoking exposure [43]. Moreover, examining whether timing, dose, or cessation of maternal smoking differentially influences T1D risk may refine our understanding of critical windows of susceptibility. It is also essential to investigate whether similar associations exist for other autoimmune conditions, such as multiple sclerosis [44], which may share pathophysiological pathways with T1D. Lastly, residual confounding remains a significant limitation of our findings. Socioeconomic, behavioral, or familial factors may plausibly explain the observed association and cannot be fully accounted for in conventional cohort analyses. Future research should incorporate negative-control exposures (such as paternal smoking) and within-family or sibling-comparison designs to better differentiate causal effects from unmeasured confounding.

Although our meta-analysis consistently indicated a modest inverse association between maternal smoking during pregnancy and the risk of T1D in offspring, this finding warrants cautious interpretation. The observed “protective” effect is counterintuitive, given the well-established adverse consequences of tobacco exposure on maternal and child health. Importantly, statistical homogeneity across studies does not eliminate the presence of clinical heterogeneity, which includes differences in exposure assessment, adjustment for confounders, follow-up periods, and population characteristics. Furthermore, residual confounding due to socioeconomic status, parental health behaviors, or unmeasured genetic–environmental interactions cannot be disregarded. Case–control and sibling-comparison designs, although methodologically distinct from prospective cohorts, have sometimes reported attenuated or null associations, underscoring the importance of study design in shaping observed results. Together, our findings contribute to the existing body of evidence but do not imply a causal protective effect of maternal smoking. Instead, they emphasize the need for further high-quality studies that meticulously control for familial, behavioral, and socioeconomic factors to clarify whether the observed association reflects a true biological mechanism, residual confounding, or methodological artifact.

## Conclusion

In conclusion, this meta-analysis of prospective cohort studies identified an inverse association between maternal smoking during pregnancy and the incidence of childhood-onset T1D in offspring. Although the association was consistent across subgroups and robust in sensitivity analyses, the counterintuitive direction of effect and the inherent limitations of observational research indicate that these results should be interpreted with caution. Residual confounding due to socioeconomic, behavioral, or genetic factors may plausibly account for the observed association. Therefore, the findings should not be construed as evidence of a causal protective effect. Instead, they underscore the complexity of prenatal influences on immune-mediated diseases and the necessity for future studies employing robust causal-inference methods to disentangle biological mechanisms from confounding. Importantly, these results do not alter the clear imperative to discourage maternal smoking during pregnancy due to its well-established adverse health consequences.

## Supplemental data

**Supplemental File 1.** **Detailed search strategy for each database**

**PubMed**

(“Pregnancy”[Mesh] OR “Pregnant Women”[Mesh] OR pregnancy[tiab] OR pregnant[tiab] OR prenatal[tiab] OR pre-natal[tiab]) AND (“Smoking”[Mesh] OR “Tobacco Use”[Mesh] OR smoking[tiab] OR smoke[tiab] OR cigarette[tiab] OR cigarettes[tiab] OR nicotine[tiab] OR tobacco[tiab]) AND (“Diabetes Mellitus, Type 1”[Mesh] OR “type 1 diabetes”[tiab] OR T1D[tiab] OR T1DM[tiab] OR diabetic[tiab] OR diabetes[tiab]) AND (“Child”[Mesh] OR “Adolescent”[Mesh] OR “Pediatrics”[Mesh] OR child[tiab] OR children[tiab] OR adolescent[tiab] OR adolescents[tiab] OR pediatric[tiab] OR paediatric[tiab] OR offspring[tiab] OR childhood[tiab] OR adolescence[tiab]) AND (humans[Filter]) AND (“journal article”[Publication Type])

**Embase**

(‘pregnancy’/exp OR ‘pregnant woman’/exp OR pregnancy:ti,ab OR pregnant:ti,ab OR prenatal:ti,ab OR pre-natal:ti,ab) AND (‘smoking’/exp OR ‘tobacco use’/exp OR smoking:ti,ab OR smoke:ti,ab OR cigarette:ti,ab OR cigarettes:ti,ab OR nicotine:ti,ab OR tobacco:ti,ab) AND (‘type 1 diabetes mellitus’/exp OR ‘diabetes mellitus’:ti,ab OR ‘type 1 diabetes’:ti,ab OR T1D:ti,ab OR T1DM:ti,ab OR diabetic:ti,ab) AND (‘child’/exp OR ‘adolescent’/exp OR ‘pediatrics’/exp OR child:ti,ab OR children:ti,ab OR adolescent:ti,ab OR adolescents:ti,ab OR pediatric:ti,ab OR paediatric:ti,ab OR offspring:ti,ab OR childhood:ti,ab OR adolescence:ti,ab) AND [humans]/lim AND [article]/lim

**Web of Science**

TS=(“pregnancy” OR “pregnant” OR “prenatal” OR “pre-natal”) AND TS=(“smoking” OR “smoke” OR “cigarette” OR “cigarettes” OR “nicotine” OR “tobacco”) AND TS=(“type 1 diabetes” OR “diabetes” OR “diabetic” OR “T1D” OR “T1DM”) AND TS=(“child” OR “children” OR “adolescent” OR “adolescents” OR “pediatric” OR “paediatric” OR “offspring” OR “childhood” OR “adolescence”) AND DT=(Article) AND LA=(English)

## Data Availability

All data generated or analyzed during this study are included in this published article.
